# Supplementary material for: Cytokines regulate the antigen-presenting characteristics of human circulating and tissue-resident intestinal ILCs
Source: Nat Commun. 2020 Apr 27;11:2049. doi: 10.1038/s41467-020-15695-x (PMC7184749; doi:10.1038/s41467-020-15695-x)
Supplement: Supplementary file 1 — Supplementary Information [file 41467_2020_15695_MOESM1_ESM.pdf]

Cytokines regulate the antigen-presenting characteristics of human circulating and tissue-resident intestinal ILCs

Rao *et al*.

Supplementary Figures

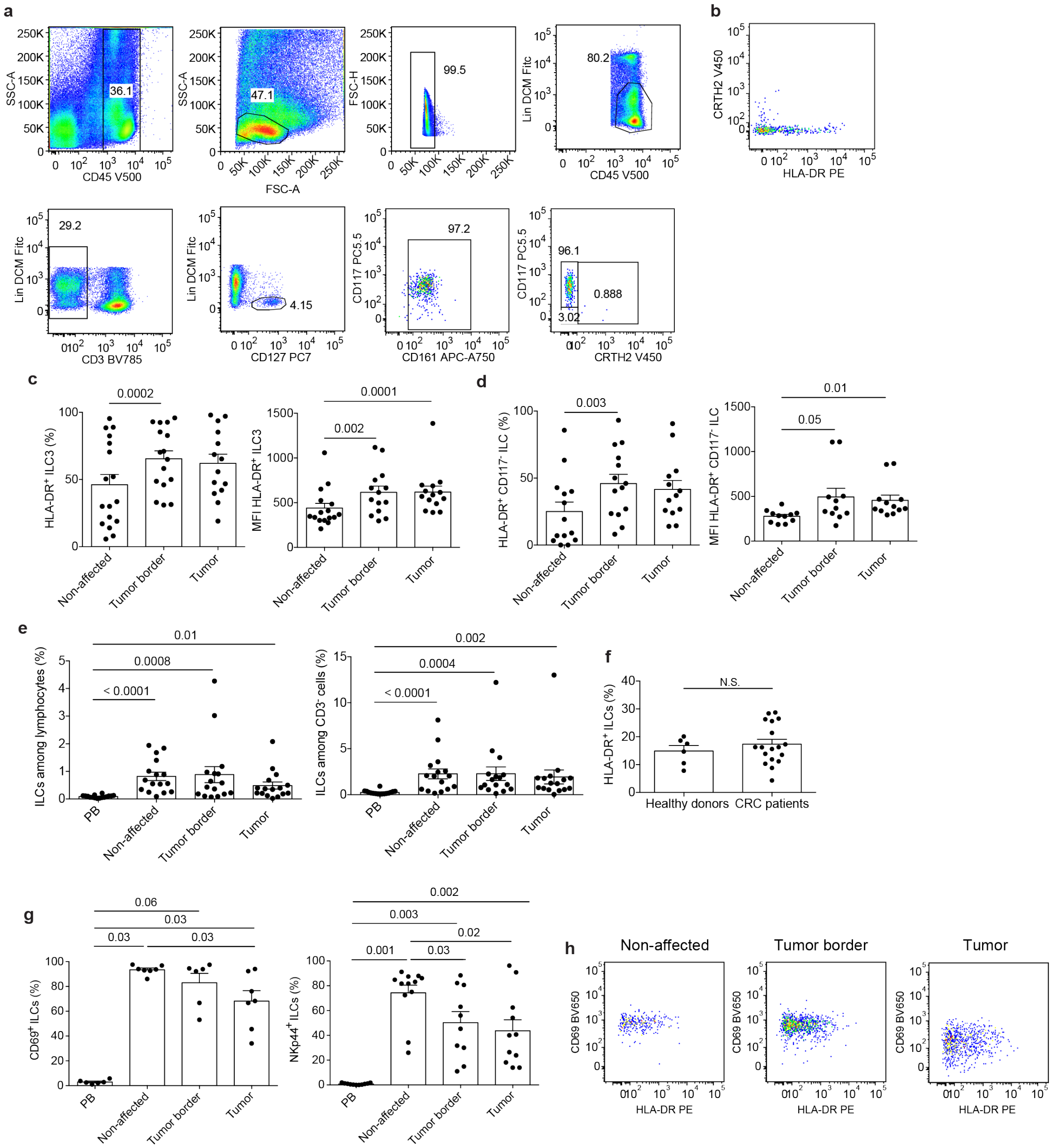

Supplementary Figure 1. (**a**) Gating strategy for assessing intestinal ILC subsets presented in Fig. 1a. Same gating strategy was used for analyzing or sorting PB and intestinal ILC subsets presented in Fig. 2a, c, Fig. 4b, d, Fig. 5a, b and Fig. 7a, as well as Supplementary Figures 1h, 2a, 2c, 5, 6c, 9, 10a, 10c, 11-13, 14a and 17b. (**b**) Representative plot of CRTH2 and HLA-DR expression on colonic CD127^{+}CD161^{+} ILCs. (**c**, **d**) Frequency and MFI of HLA-DR expression

on (**c**) ILC3 and (**d**) CD117$^-$ ILCs from non-affected colon, tumor border and central tumor tissues; N (patients, ILC3) = 17 (frequency) and 16 (MFI); N (patients, CD117$^-$ ILCs) = 15 (frequency) and 12 (MFI). Not all sub-anatomical regions could be obtained for every donor. Bars and error bars indicate mean and SEM; statistical significance was assessed using two-sided Wilcoxon matched-pairs signed rank test. (**e**) Frequency of ILCs among total CD45$^+$ lymphocytes and CD3$^-$CD45$^+$ lymphocytes in PB, non-affected colon, tumor border and central tumor tissues; N (patients) = 16; bars and error bars indicate mean and SEM; statistical significance was assessed using Friedman test and Dunn's multiple comparison test. (**f**) Frequency of HLA-DR$^+$ ILCs in PB of healthy individuals (N = 6) and patients with CRC (N = 18); bars and error bars indicate mean and SEM; statistical significance was assessed using two-sided Wilcoxon matched-pairs signed rank test. (**g**) CD69 and NKp44 expression on ILCs from PB, non-affected colon, tumor border and central tumor tissues; N (patients) = 7 (for CD69) and 12 (for NKp44). Not all sub-anatomical regions could be obtained for every donor. Bars and error bars indicate mean and SEM; statistical significance was assessed using two-sided Wilcoxon matched-pairs signed rank test. (**h**) Representative plot of CD69 and HLA-DR expression on colonic CD127$^+$CD161$^+$ ILCs from different sub-anatomical regions. Source data are provided as a source data file.

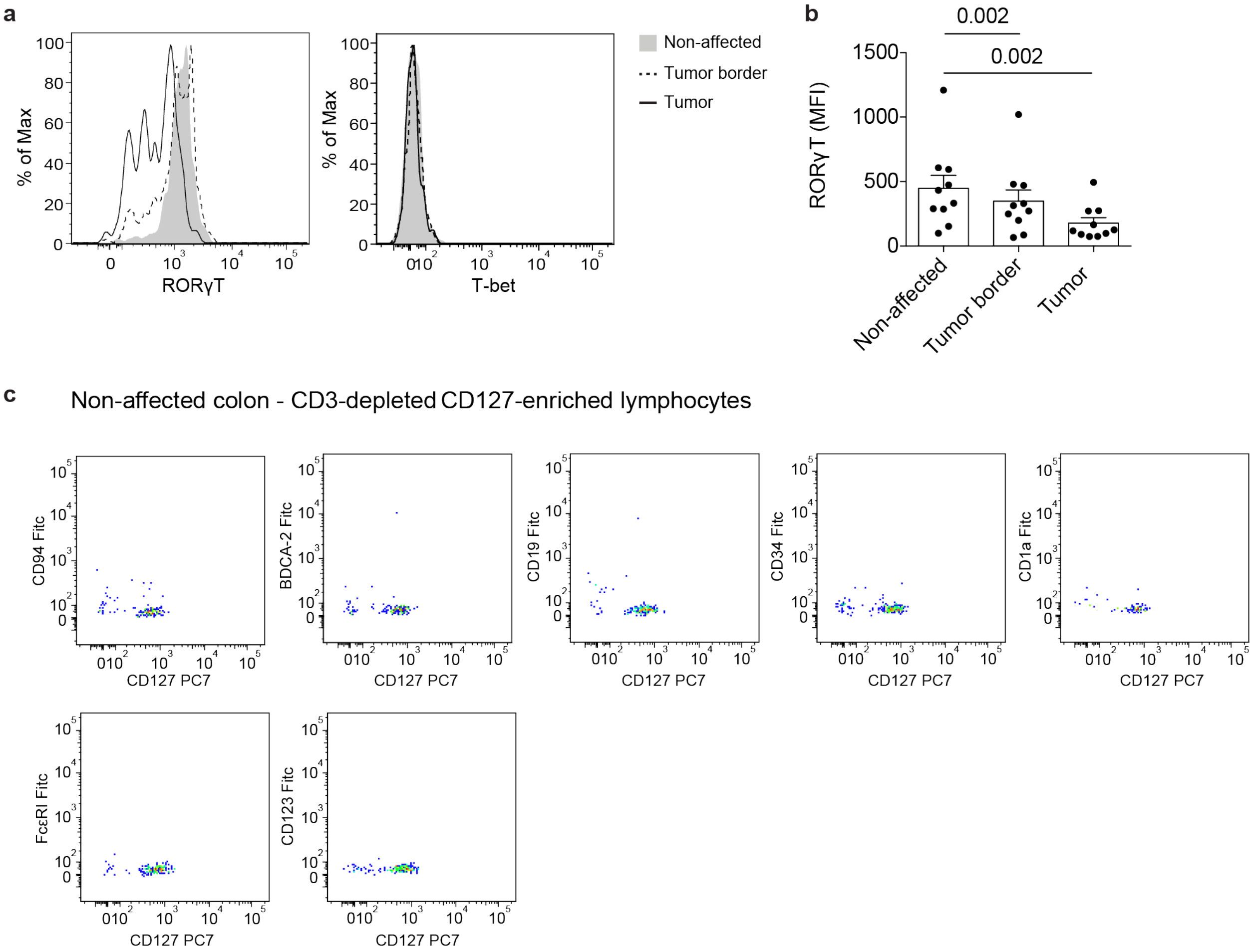

Supplementary Figure 2. (**a**) Histograms of RORγT and T-bet expression in colon-derived ILCs from different sub-anatomical areas; representative plots of one out of 10 analyzed donors are depicted. (**b**) Mean fluorescence intensity of RORγT expression in colon-derived ILCs; N (patients) = 10; bars and error bars indicate mean and SEM; statistical significance was assessed using two-sided Wilcoxon matched-pairs signed rank test. Source data are provided as a source data file. (**c**) Lineage marker expression on CD127$^+$CD3$^-$ lymphocytes from non-affected colon tissue. Colonic mononuclear cells were magnetically depleted for CD3 and enriched for CD127-expressing cells prior to flow cytometric analysis. CD3$^+$ cell-contamination was excluded by CD3 staining of the purified fraction. Depicted are CD3$^-$ lymphocytes.

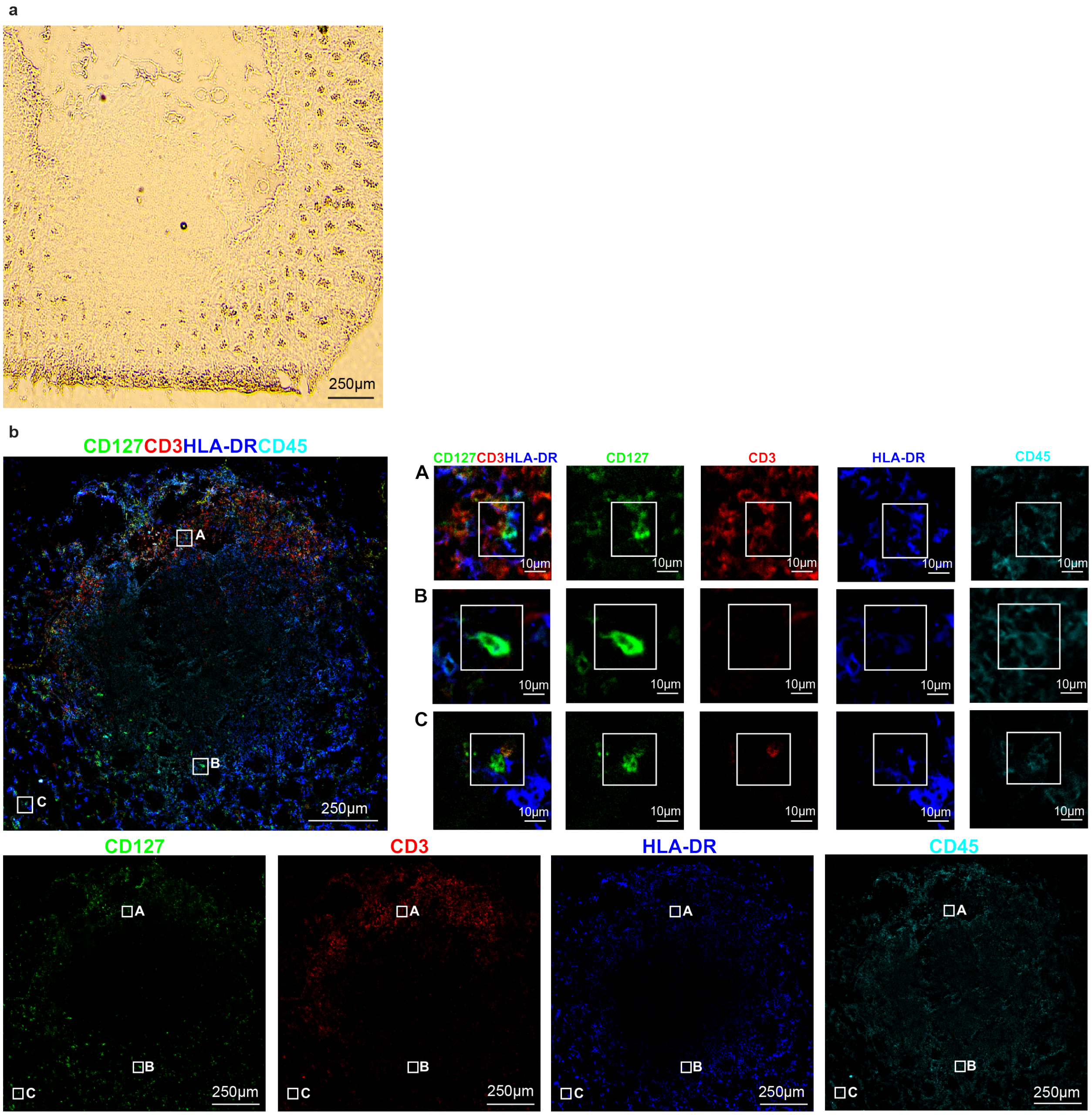

Supplementary Figure 3. Lymphoid follicle-containing region of non-affected colon acquired by (**a**) light and (**b**) multicolor immunofluorescence microscopy. Depicted are the distribution and HLA-DR expression of $CD127^{+}CD3^{-}CD45^{+}$ ILCs and $CD3^{+}$ T cells, respectively. Magnified regions show examples of (A) $HLA\text{-}DR^{+}CD127^{+}$ ILCs in contact with $CD3^{+}$ T cells, (B) $HLA\text{-}DR^{+}CD127^{+}$ ILCs alone and (C) $CD127^{+}$ ILCs co-localized with $HLA\text{-}DR^{hi}CD45^{+}$ cells and $CD3^{+}$ T cells; individual channels are shown separately. Representative of 3 patients analyzed in 3 independent experiments.

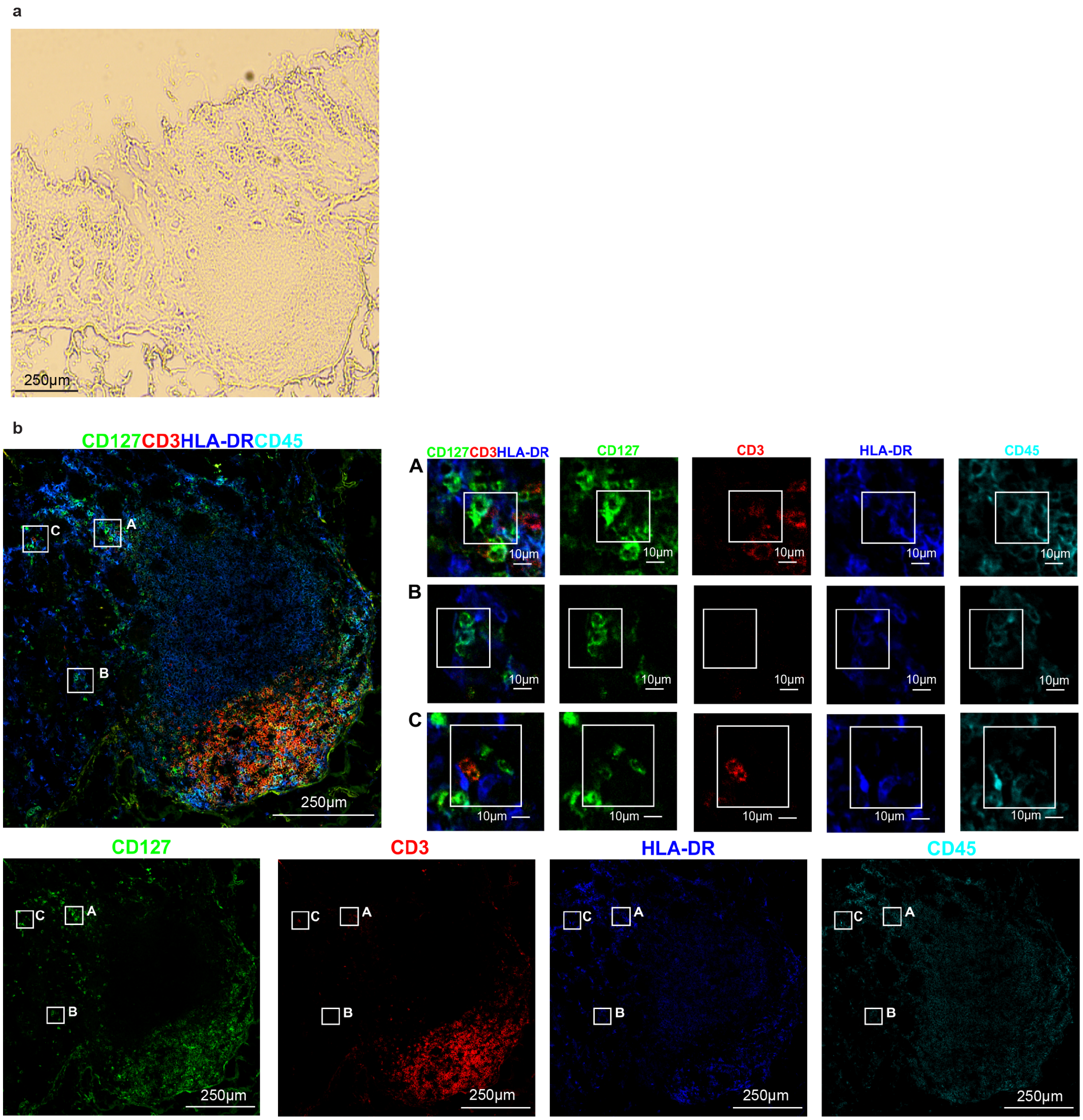

Supplementary Figure 4. Lymphoid follicle-containing region of colorectal tumor border tissue acquired by (**a**) light and (**b**) multicolor immunofluorescence microscopy. Depicted are the distribution and HLA-DR expression of CD127$^+$CD3$^-$CD45$^+$ ILCs and CD3$^+$ T cells, respectively. Magnified regions show examples of (A) HLA-DR$^+$CD127$^+$ ILCs in contact with CD3$^+$ T cells, (B) HLA-DR$^+$CD127$^+$ ILCs alone and (C) CD127$^+$ ILCs co-localized with HLA-DR^{hi}CD45$^+$ cells and CD3$^+$ T cells; individual channels are shown separately. Representative of 3 patients analyzed in 3 independent experiments.

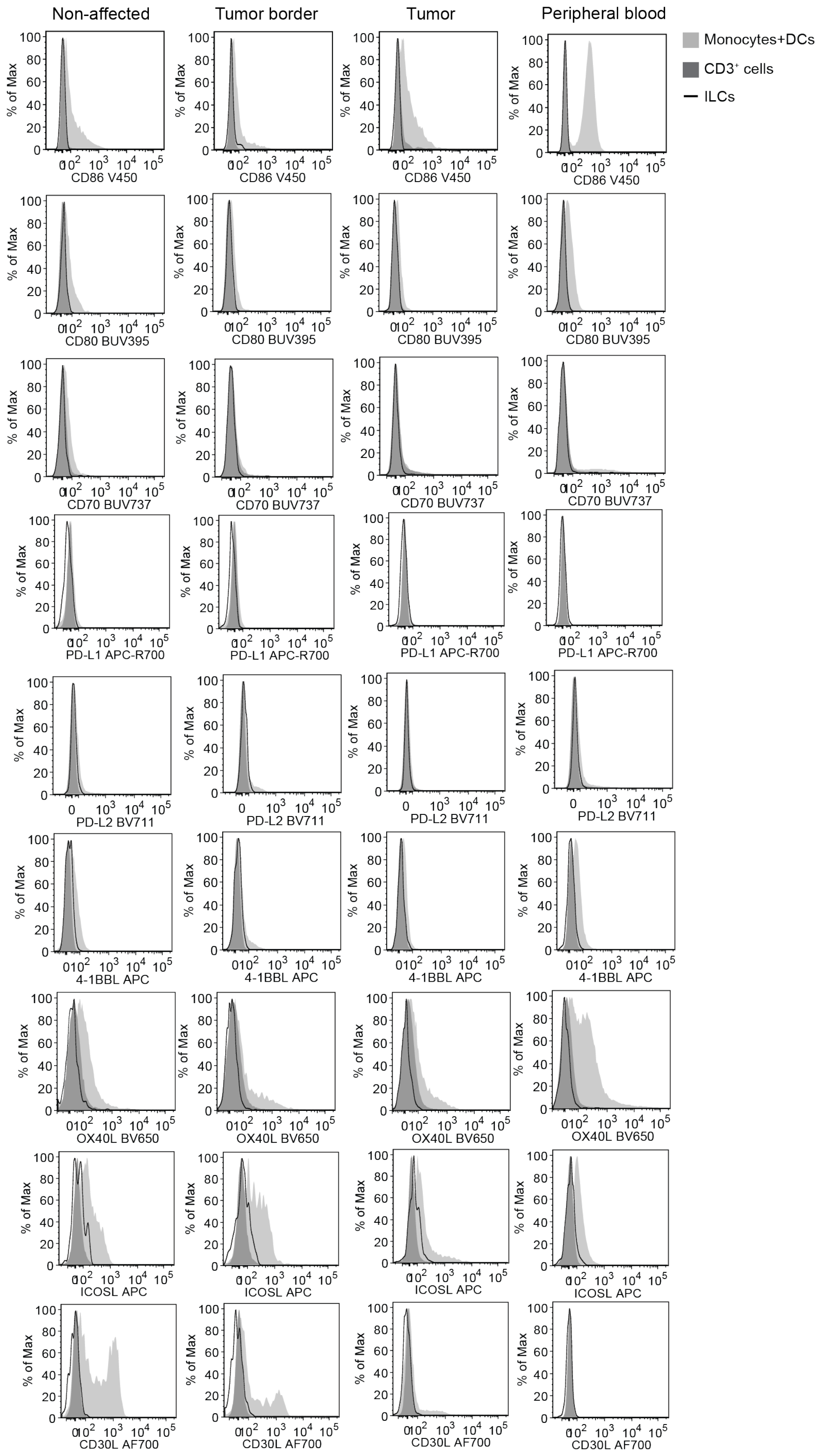
Non-affected
Tumor border
Tumor
Peripheral blood
Monocytes+DCs
CD3+ cells
ILCs
% of Max
CD86 V450
CD80 BUV395
CD70 BUV737
PD-L1 APC-R700
PD-L2 BV711
4-1BBL APC
OX40L BV650
ICOSL APC
CD30L AF700

Supplementary Figure 5. Co-stimulatory molecule expression on ILCs from PB, non-affected, tumor border and central tumor tissue of the colon. Representative overlay histograms of CD86, CD80, CD70, PD-L1, PD-L2, 4-1BBL, OX40L, ICOSL and CD30L expression on monocytes plus DCs (light grey), $CD3^+$ T cells (dark grey) and ILCs (black line).

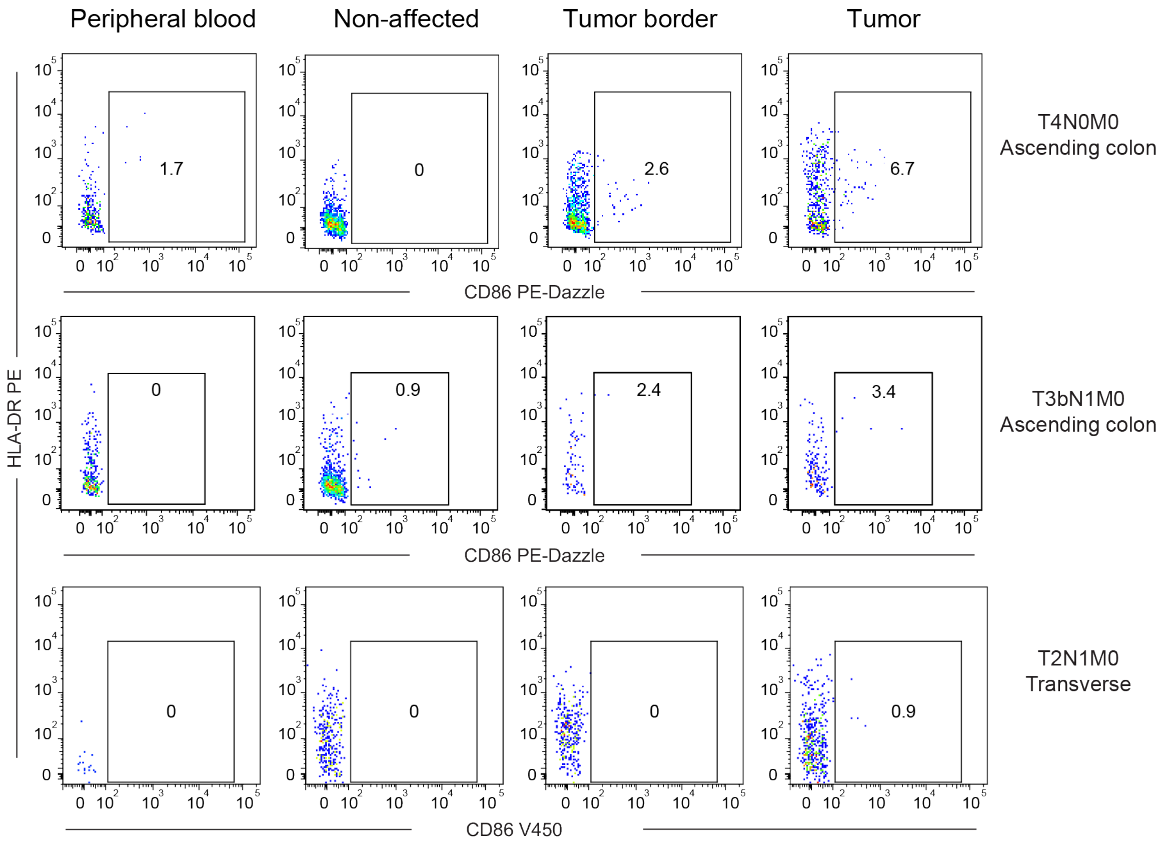

Supplementary Figure 6. Various degrees of CD86 expression detected on ILCs from 3 out of 13 patients with CRC analyzed.

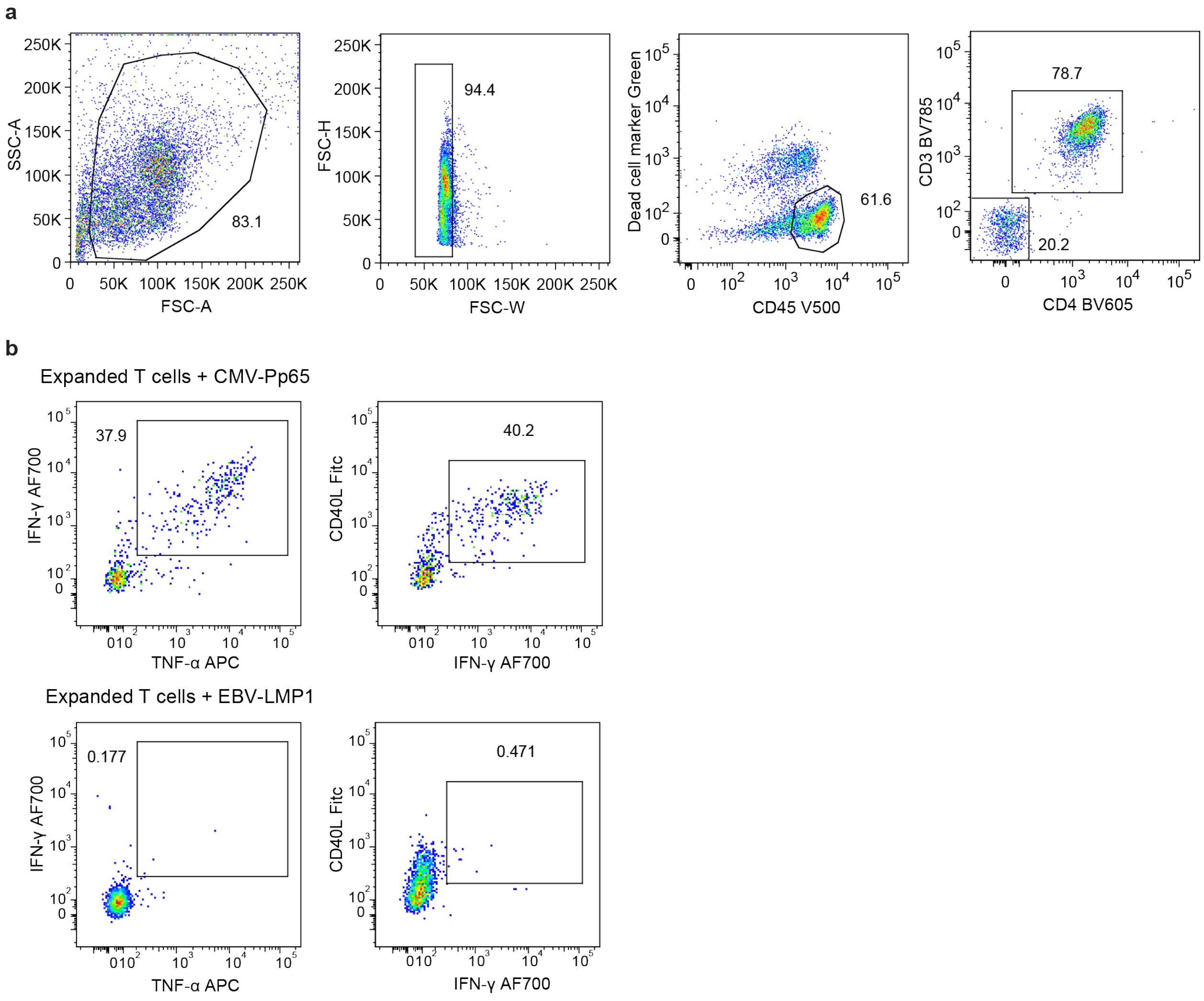

Supplementary Figure 7. (**a**) Gating strategy for assessing T-cell response depicted in Fig. 3b, Fig. 6b and Supplementary Figure 7b, and for analyzing HLA-DR and CD40 expression on antigen presenting cells shown in Fig. 3d and Supplementary Figure 17b. (**b**) Specificity of expanded CMV-pp65-specific CD4$^+$ memory T cells. IFN-γ and TNF-α production as well as CD40L expression of enriched and expanded CMV-pp65-specific CD4$^+$ memory T cells following stimulation with CMV-pp65 or EBV-LMP1 peptide pools.

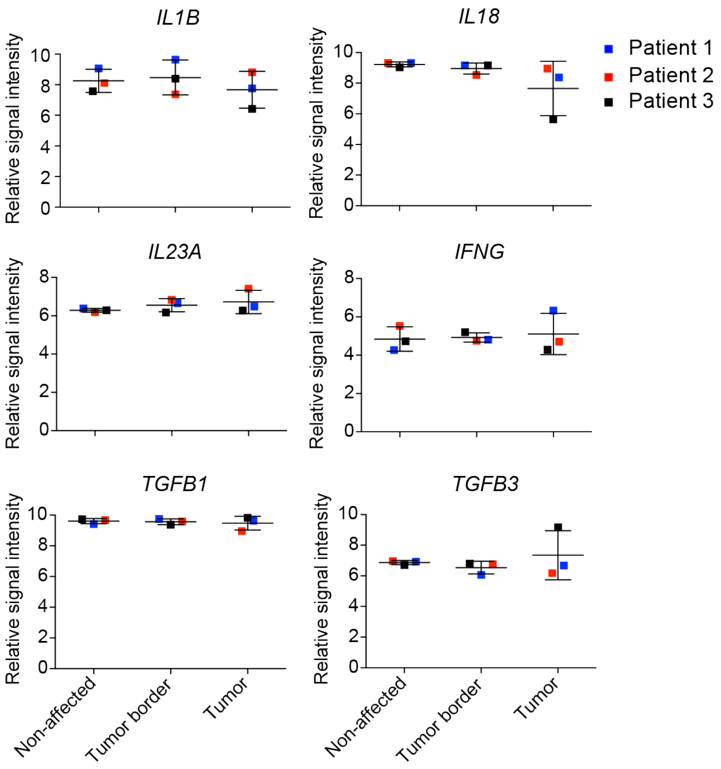

Supplementary Figure 8. Relative signal intensity ± SD of *IL1B*, *IL18*, *IL23A*, *IFNG*, *TGFB1* and *TGFB3* gene transcripts in non-affected colon, tumor border and central tumor areas from three patients with CRC. Source data are provided as a source data file.

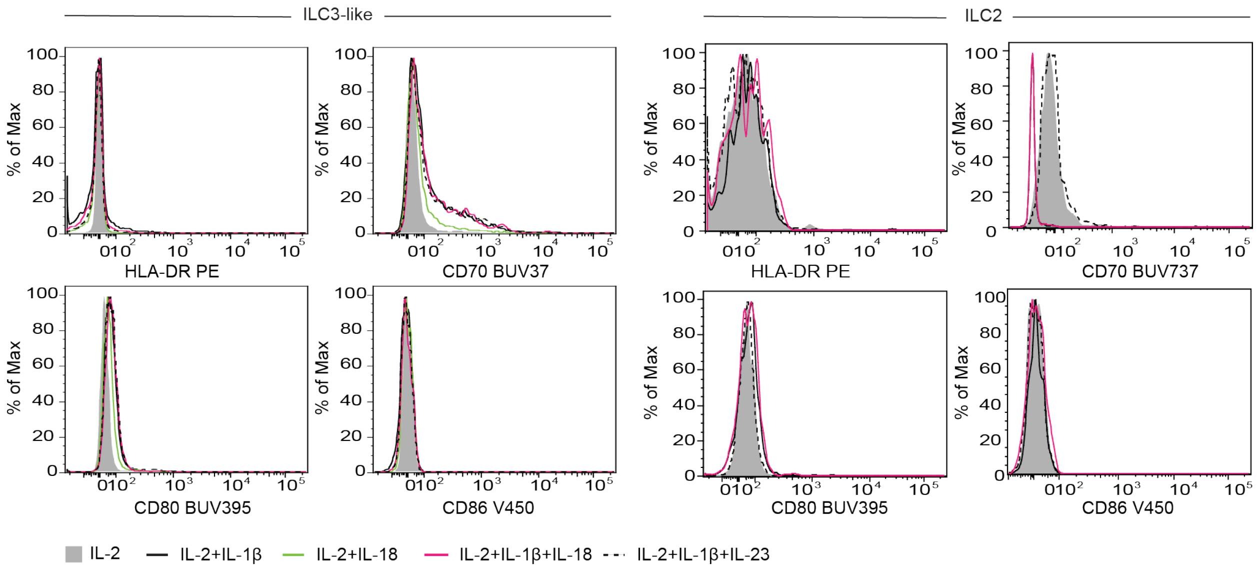

Supplementary Figure 9. HLA-DR and co-stimulatory molecule expression on sort-purified PB ILC2 and ILC3-like cells after 24h of treatment with indicated cytokine combinations.

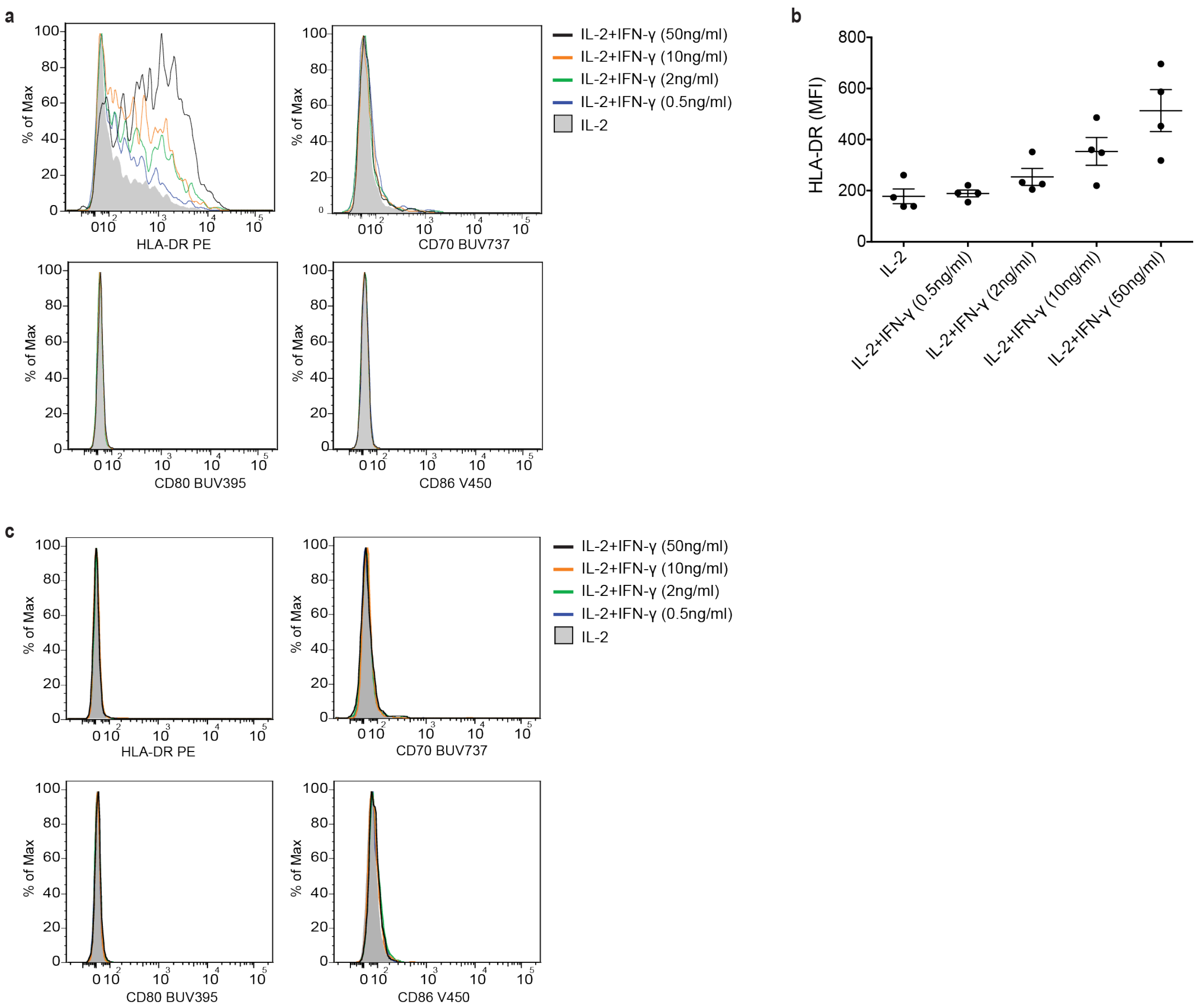

Supplementary Figure 10. Dose response of PB ILC subsets to IFN-γ stimulation. HLA-DR and co-stimulatory molecule expression was assessed on sort-purified (**a**, **b**) ILC3-like cells and (**c**) ILC2 following 5 days of stimulation. (**b**) N (donors) = 4. Mean +/- SEM is displayed. Source data are provided as a source data file.

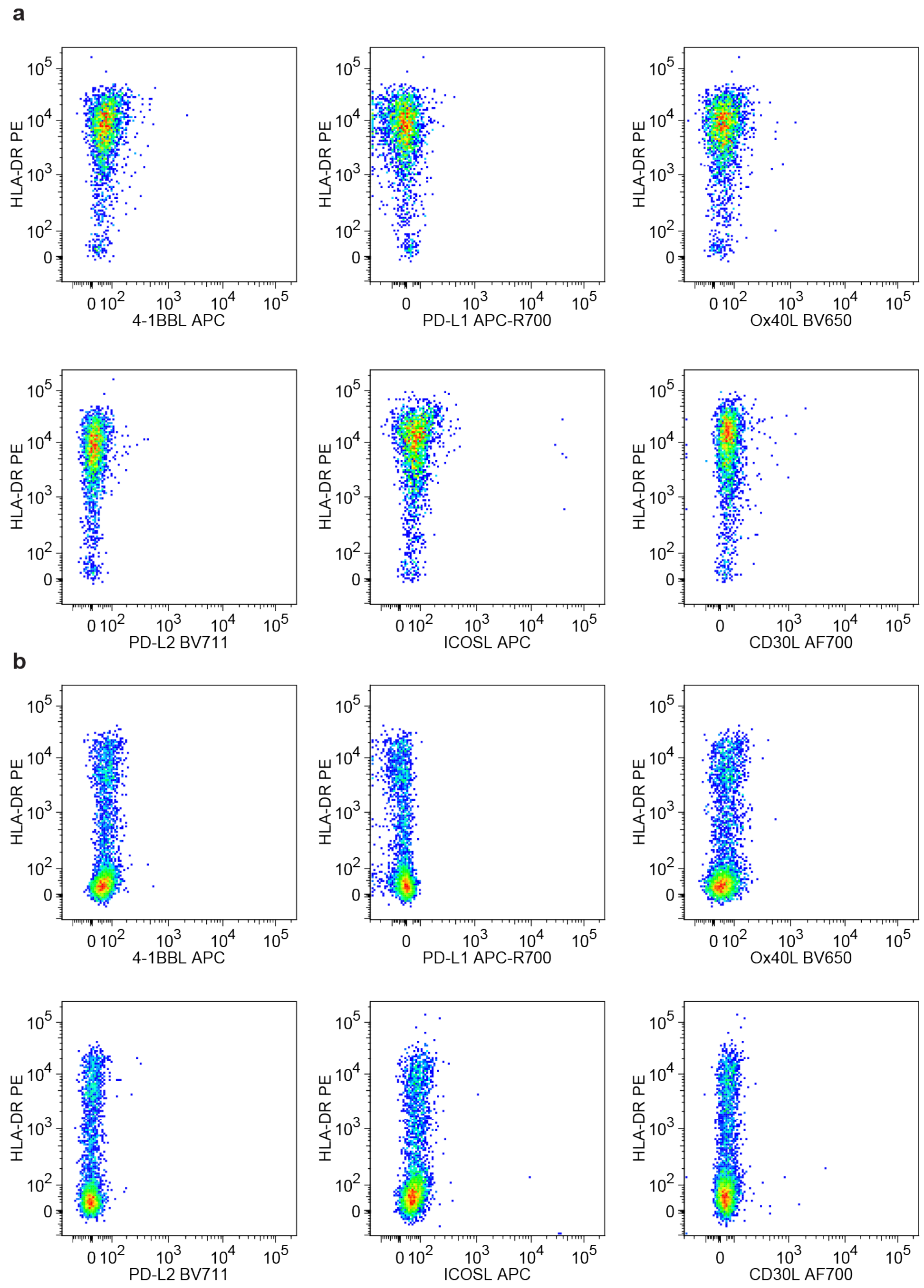

Supplementary Figure 11. 4-1BBL, PD-L1, PD-L2, OX40L, ICOSL and CD30L expression on sort-purified PB (**a**) ILC3-like cells and (**b**) ILC2 after 5 days stimulation with IL-2 plus IL-1β.

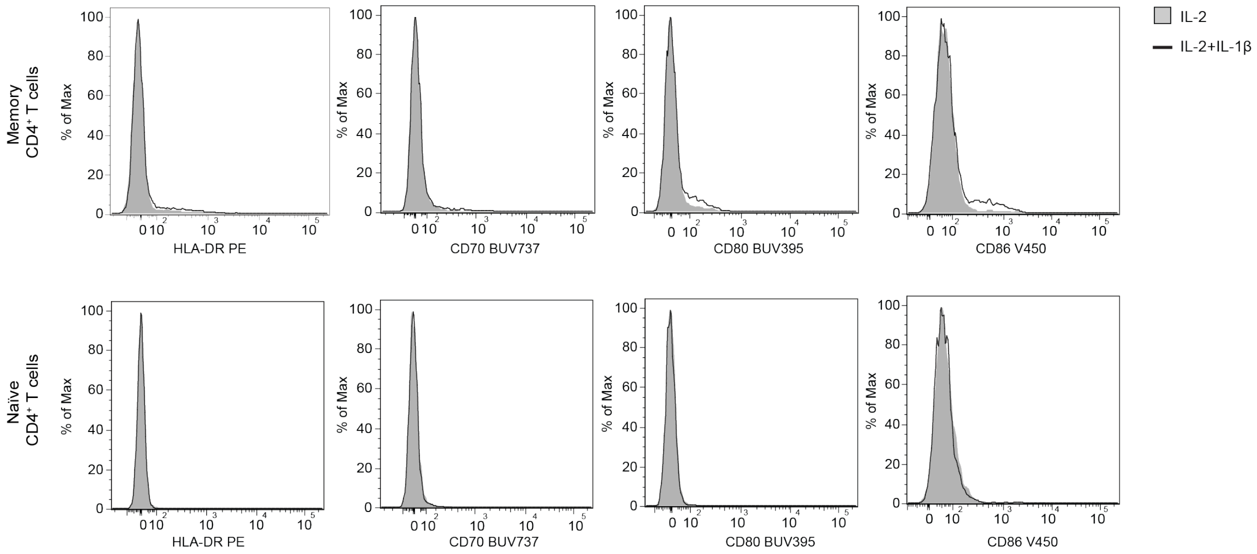

Supplementary Figure 12. Overlay histograms of HLA-DR and co-stimulatory molecule expression on sort-purified PB memory and naïve CD4$^+$ T cells after 5 days stimulation with IL-2 (grey) or IL-2 plus IL-1β (black line).

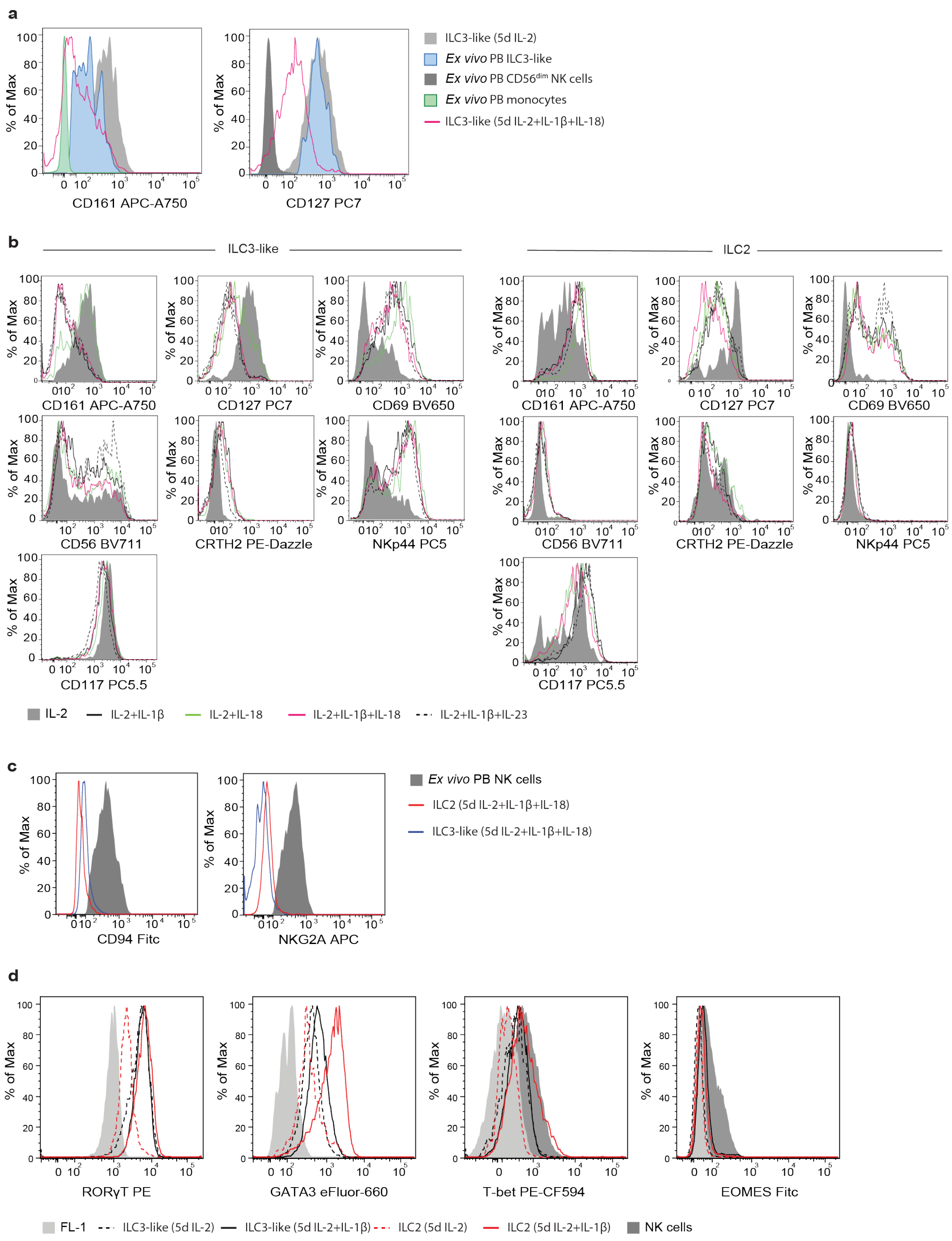

Supplementary Figure 13. (**a**) CD127 and CD161 expression on *ex vivo* ILC3-like cells and ILC3-like cells following 5 days culture with IL-2 or IL-2 plus IL-1β and IL-18. *Ex vivo* PB monocytes and CD56^{dim} NK cells were used as negative controls for CD161 and CD127 expression, respectively. (**b**) Surface marker expression of sort-purified PB ILC2 and ILC3-like

cells after 5 days treatment with indicated cytokine combinations. (**c**) CD94 and NKG2A expression on sort-purified PB ILC2 and ILC3-like cells following 5 days culture with IL-2, IL-1β and IL-18. (**d**) Representative histograms of RORγT, GATA3, T-bet and EOMES expression by PB ILC2 and ILC3-like cells following 5 days treatment with the indicated cytokine combinations. FL-1 depicts fluorescence minus one control.

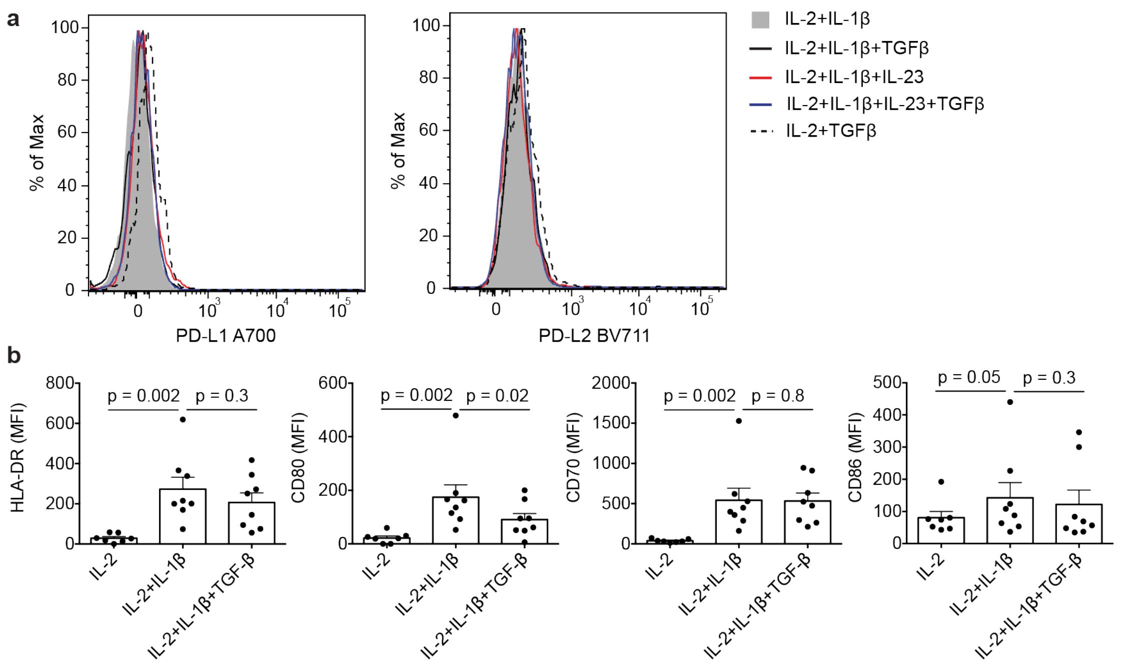

Supplementary Figure 14. (**a**) PD-L1 and PD-L2 expression on PB ILC3-like cells following 5 days culture with indicated cytokine combinations. (**b**) HLA-DR, CD86, CD80 and CD70 expression by sort-purified ILC2 after 5 days of IL-2, IL-2 plus IL-1β, or IL-2 plus IL-1β and TGF-β treatment; N (donors) = 8. Due to limited cell numbers, we could not generate data for all conditions for every donor; source data are provided as a source data file. Bars and error bars indicate mean and SEM; statistical significance was assessed using two-sided Wilcoxon matched-pairs signed rank test. Source data are provided as a source data file.

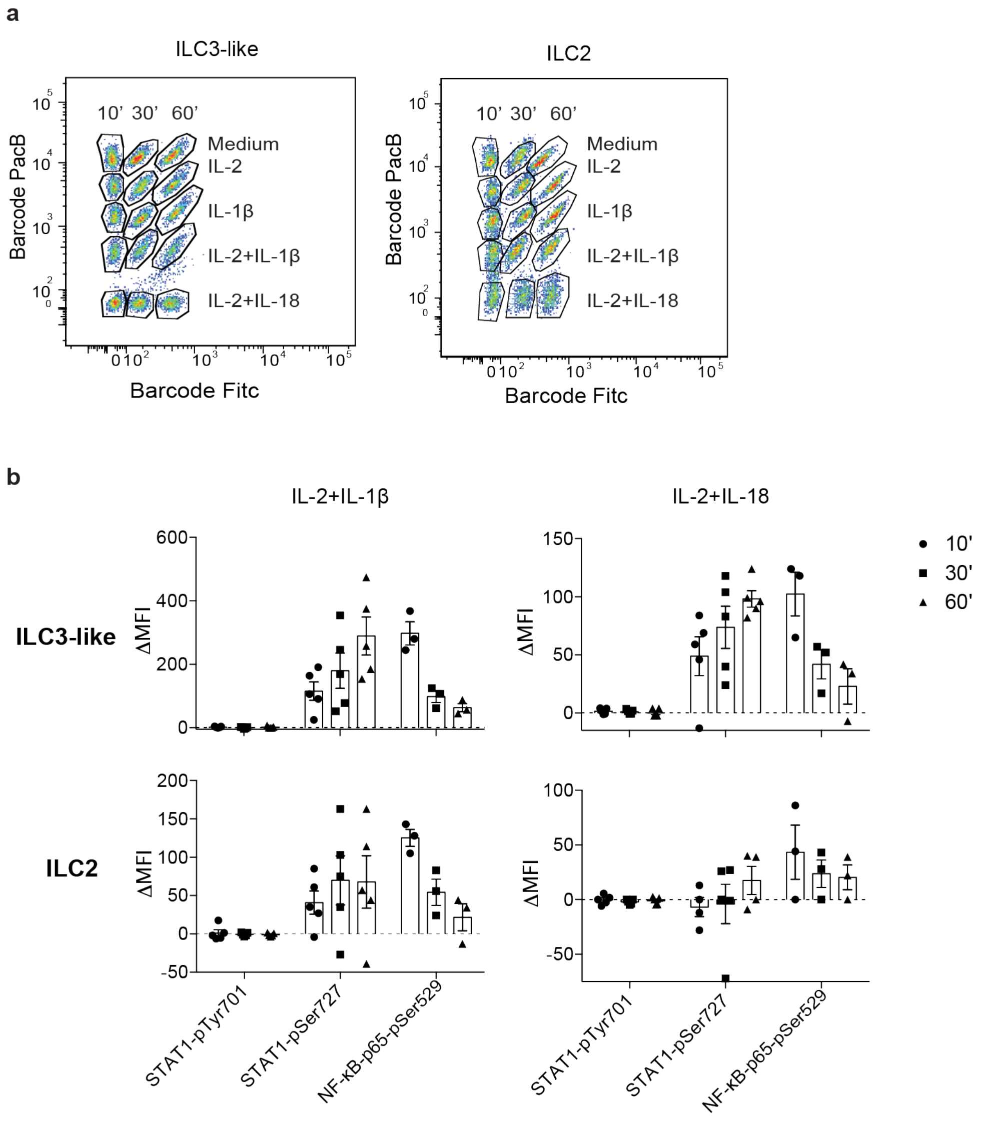

Supplementary Figure 15. (**a**) Gating strategy of barcoded, stimulated PB ILC2 and ILC3-like cells during phospho-flow analysis. (**b**) Mean values of ΔMFI $STAT1_{Tyr701}$, $STAT1_{Ser727}$ and NF-κB-$p65_{Ser529}$ phosphorylation in sorted PB ILC2 and ILC3-like cells following 10-, 30- or 60-minute exposure to IL-2 plus IL-1β or IL-2 plus IL-18. Data was normalized to background MFI. N (donors) = 3 for NF-κB and 5 for STAT1 phosphorylation; Error bars represent SEM. Source data are provided as a source data file.

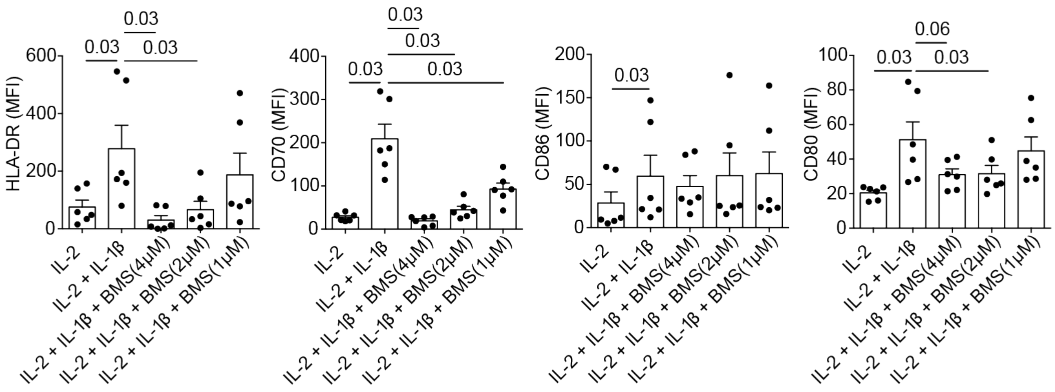

Supplementary Figure 16. MFI of HLA-DR, CD70, CD80 and CD86 expression on sorted PB ILC3-like cells, following 72 hours incubation with IL-2, IL-2 plus IL-1β, or IL-2 plus IL-1β in the presence of various concentrations of BMS-345541. N (donors) = 6; bars and error bars indicate mean and SEM; statistical significance was assessed using two-sided Wilcoxon matched-pairs signed rank test. Source data are provided as a source data file.

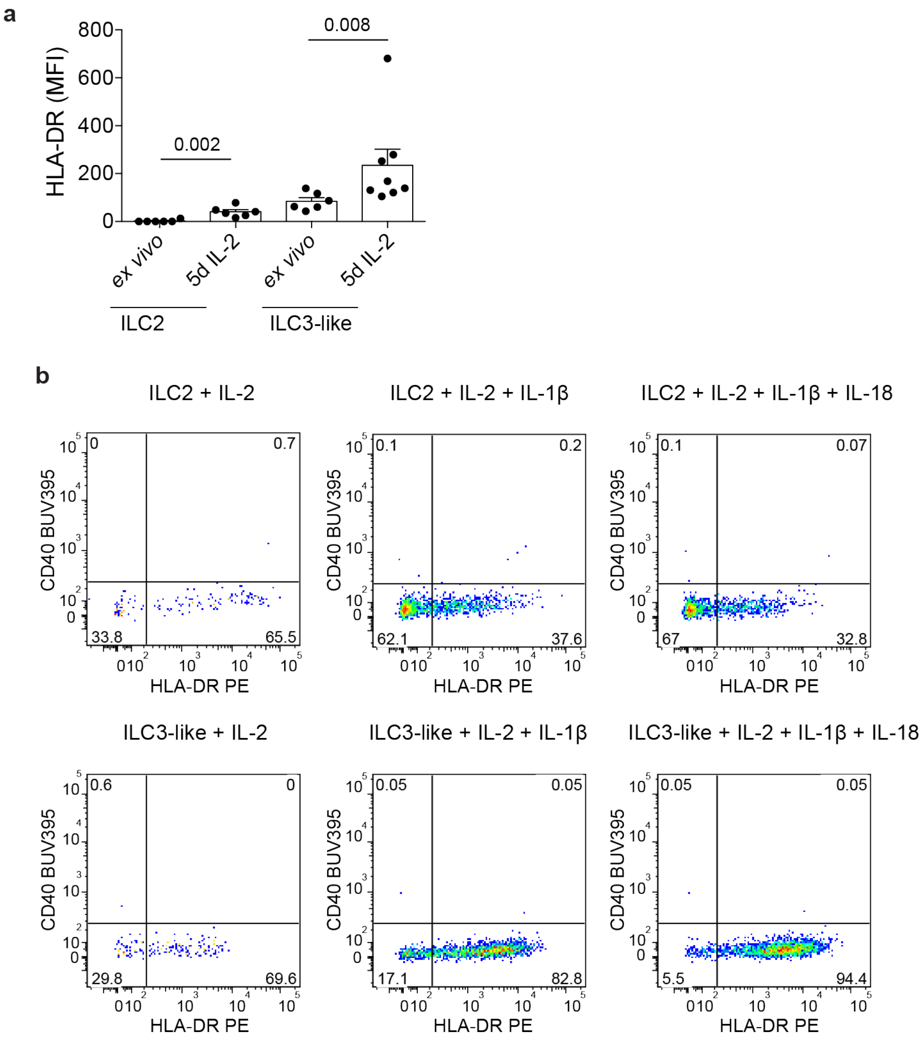

Supplementary Figure 17. (**a**) HLA-DR expression on PB ILC2 and ILC3-like cells *ex vivo* or following 5-day culture with 10U/ml IL-2. N (donors) = 6 for *ex vivo* ILC2 and ILC3-like cells, 6 for expanded ILC2 and 8 for expanded ILC3-like cells; bars and error bars indicate mean and SEM; statistical significance was assessed using two-sided Mann-Whitney *U* test. Source data are provided as a source data file. (**b**) Representative plots of HLA-DR and CD40 expression on stimulated sort-purified PB ILC subsets present in the autologous CMV-pp65-specific co-culture system.

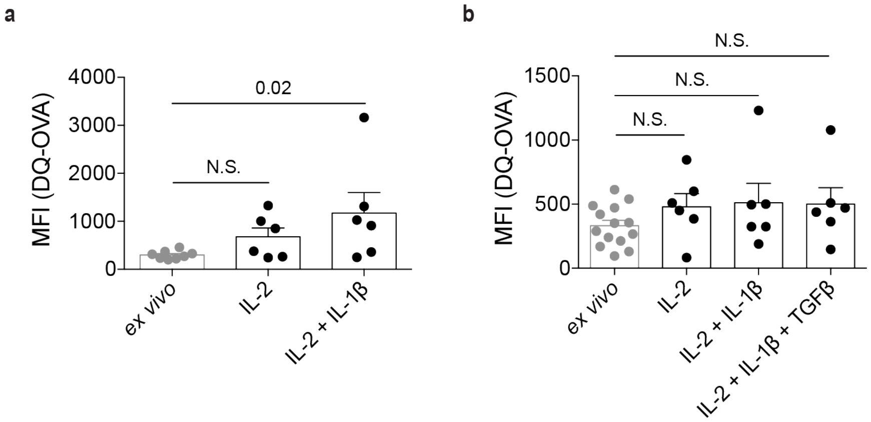

Supplementary Figure 18. Fluorescence emission after 4h incubation with 10μg/ml DQ-OVA by PB ILC2 (**a**) or ILC3-like cells (**b**) isolated *ex vivo* (N donors = 8 for ILC2 and 14 for ILC3-like cells) or expanded for 5 days with the indicated cytokine combinations (N donors = 6); Bars and error bars represent mean and SEM; statistical significance was assessed using Mann-Whitney *U* test. Source data are provided as a source data file.

Supplementary Tables

Table S1. Patient information:

Number	Age	Gender	Tumor location	Tumor stage
#1	61	m	Rectum	II
#2	64	f	Transverse	II
#3	78	f	Sigmoid	III
#4	60	f	Sigmoid	III
#5	70	m	Ascending	II
#6	75	f	Ascending	III
#7	72	f	Sigmoid	III
#8	79	m	Sigmoid	II
#9	70	m	Sigmoid	III
#10	76	m	Cecum	II
#11	53	m	Sigmoid	II
#12	87	f	Transverse	IV
#13	71	f	Cecum	II
#14	60	f	Transverse	III
#15	75	f	Ascending	II
#16	72	m	Ascending	II
#17	75	m	Ascending	II
#18	41	f	Sigmoid	IV
#19	84	m	Sigmoid	II
#20	81	m	Sigmoid	IV
#21	76	m	Ascending	II
#22	65	f	Cecum	III
#23	48	m	Cecum	II
#24	54	f	Ascending	II
#25	91	f	Transverse	III
#26	81	m	Right colic flexure	III
#27	74	m	Descending	II

Table S2. Full list of antibodies used for flow-cytometric analysis and sorting:

Lineage

Reactivity	Clone	Fluorophore	Catalog #	Dilution	Company
CD14	TÜK4	Fitc	F0844	2:50	Dako
CD1a	HI149	Fitc	300103	2:50	Biolegend
CD123	6H6	Fitc	306013	2:50	Biolegend
FcεRIα	AER-37	Fitc	334607	2:50	Biolegend
TCRα/β	IP26	Fitc	306705	2:50	Biolegend
TCRγ/δ	B1	Fitc	331208	2:50	Biolegend
CD94	DX22	Fitc	305504	2:50	Biolegend
BDCA2	AC144	Fitc	130-113-192	2:50	Miltenyi Biotec
CD19	4G7	Fitc	392507	2:50	Biolegend
CD34	581	Fitc	343504	2:50	Biolegend

Surface staining

Reactivity	Clone	Fluorophore	Catalog #	Dilution	Company
CD3	OKT3	BV785	317329	1:50	Biolegend
CRTH2	BM16	V450	561661	4:50	BD Biosciences
CRTH2	BM16	PE-Dazzle	350125	4:50	Biolegend
CD45	HI30	V500	560777	1:50	BD Biosciences
CD161	191B8	APC-A750	B30630	3:50	Beckman Coulter
CD117	104D2D1	PC5.5	A66333	3:50	Beckman Coulter
CD127	R34.34	PC7	A64618	3:50	Beckman Coulter
CD127	A019D5	PE-Cy5	351323	2:50	Biolegend
HLA-DR	L243	PE	307605	1:50	Biolegend
CD80	L307.4	BUV395	565210	2:50	BD Biosciences
CD70	Ki-24	BUV737	565339	2:50	BD Biosciences
CD86	FUN-1	V450	560357	2:50	BD Biosciences
CD86	IT2.2	PE-Dazzle	305433	2:50	Biolegend
PDL1	MIH1	APC-R700	565188	3:50	BD Biosciences
PDL2	MIH18	BV711	740818	2:50	BD Biosciences
4-1BBL	5F4	APC	311505	2:50	Biolegend
OX40L	11C3.1	Biotin	326306	2:50	Biolegend

Streptavidin	N.A.	BV650	405231	1:50	Biolegend
CD30L	116614	AF700	FAB1028N-100UG	2:50	R&D Systems
ICOSL	MIH12	APC	130-098-738	1:50	Miltenyi
CD4	OKT4	APC-Cy7	317417	2:50	Biolegend
CD56	HCD56	BV711	318336	2:50	Biolegend
NKp44	Z231	PC5	A66903	3:50	Beckman Coulter
CD69	FN50	BV650	310934	2:50	Biolegend
CD40	5C3	BUV395	565202	1:50	BD Biosciences
NKG2a	Z199	APC	A60797	2:50	Beckman Coulter

Intracellular Staining

**Reactivity**	**Clone**	**Fluorophore**	**Catalog #**	**Dilution**	**Company**
CD40L (CD154)	5C8	Fitc	130-100-354	1:50	Miltenyi Biotec
CD40L (CD154)	5C8	PE-Vio770	130-114-134	1:50	Miltenyi Biotec
TNF-α	Mab11	APC	502913	1:100	Biolegend
IFN-γ	B27	AF700	557995	1:100	BD Biosciences

Transcription factor Staining

**Reactivity**	**Clone**	**Fluorophore**	**Catalog #**	**Dilution**	**Company**
RORγT	Q21-559	PE	563081	1:50	BD Biosciences
T-bet	04-46	PE-CF594	562467	1:50	BD Biosciences
GATA3	TWAJ	eFluor-660	50-9966-42	1:50	eBioscience
EOMES	WD1928	Fitc	11-4877-42	1:50	eBioscience

Phospho-flow

**Reactivity**	**Clone**	**Fluorophore**	**Catalog #**	**Dilution**	**Company**
STAT1-pTyr701	4a	AF647	612597	1:20	BD Biosciences
STAT1-pSer727	A15158B	PE	686403	1:20	Biolegend
NF-κB p65- pS529	K10-895.12.50	PE-CF594	565447	1:200	BD Biosciences
