# Supplementary material for: Cytokines regulate the antigen-presenting characteristics of human circulating and tissue-resident intestinal ILCs
Source: Nat Commun. 2020 Apr 27;11:2049. doi: 10.1038/s41467-020-15695-x (PMC7184749; doi:10.1038/s41467-020-15695-x)
Supplement: Supplementary file 3 — Reporting Summary [file 41467_2020_15695_MOESM3_ESM.pdf]

nature research

Corresponding author(s): Jenny Mjösberg, Anna Rao

Last updated by author(s): Mar 3, 2020

Reporting Summary

Nature Research wishes to improve the reproducibility of the work that we publish. This form provides structure for consistency and transparency in reporting. For further information on Nature Research policies, see Authors & Referees and the Editorial Policy Checklist.

Statistics

For all statistical analyses, confirm that the following items are present in the figure legend, table legend, main text, or Methods section.

n/a	Confirmed	
☐	☒	The exact sample size (*n*) for each experimental group/condition, given as a discrete number and unit of measurement
☐	☒	A statement on whether measurements were taken from distinct samples or whether the same sample was measured repeatedly
☐	☒	The statistical test(s) used AND whether they are one- or two-sided<br>*Only common tests should be described solely by name; describe more complex techniques in the Methods section.*
☒	☐	A description of all covariates tested
☒	☐	A description of any assumptions or corrections, such as tests of normality and adjustment for multiple comparisons
☐	☒	A full description of the statistical parameters including central tendency (e.g. means) or other basic estimates (e.g. regression coefficient) AND variation (e.g. standard deviation) or associated estimates of uncertainty (e.g. confidence intervals)
☐	☒	For null hypothesis testing, the test statistic (e.g. *F*, *t*, *r*) with confidence intervals, effect sizes, degrees of freedom and *P* value noted<br>*Give P values as exact values whenever suitable.*
☒	☐	For Bayesian analysis, information on the choice of priors and Markov chain Monte Carlo settings
☒	☐	For hierarchical and complex designs, identification of the appropriate level for tests and full reporting of outcomes
☐	☒	Estimates of effect sizes (e.g. Cohen's *d*, Pearson's *r*), indicating how they were calculated

Our web collection on statistics for biologists contains articles on many of the points above.

Software and code

Policy information about availability of computer code

Data collection	FACS data was collected using BD FACSDiva software (version 8.0). Microscopy data was acquired using Nikon NIS-Elements software (version 5.02).
Data analysis	Graphpad Prism 6 for Mac and Graphpad Prism 8 for Mac.<br>R version 3.6. and Bioconductor version 3.9 (BiocManager 1.30.10) for microarray analysis of gene expression; Following packages were used: oligo version 1.48.0, affycoretools 1.56.0, ggplot2_3.2.1, gplots 3.0.1.2, clariomdhumantranscriptcluster.db 8.7.0.<br>Microscopy images were generated using Cytosketch build 310 (CytoCode). Quantification of microscopy findings was undertaken using Imaris (Version 9.3, Oxford Instruments).<br>Flow cytometry analysis was performed using FlowJo v. 9.9 (TreeStar) and SPICE software (version 5.35).

For manuscripts utilizing custom algorithms or software that are central to the research but not yet described in published literature, software must be made available to editors/reviewers. We strongly encourage code deposition in a community repository (e.g. GitHub). See the Nature Research guidelines for submitting code & software for further information.

Data

Policy information about availability of data

All manuscripts must include a data availability statement. This statement should provide the following information, where applicable:

- Accession codes, unique identifiers, or web links for publicly available datasets
- A list of figures that have associated raw data
- A description of any restrictions on data availability

The data that support the findings of this study are available from the corresponding authors upon reasonable request. Affymetrix microarray data generated during the current study are available on the Gene Expression Omnibus (GEO) database under the accession number GSE145626 [https://www.ncbi.nlm.nih.gov/geo/query/acc.cgi?acc=GSE145626].

Field-specific reporting

Please select the one below that is the best fit for your research. If you are not sure, read the appropriate sections before making your selection.

☒ Life sciences ☐ Behavioural & social sciences ☐ Ecological, evolutionary & environmental sciences

For a reference copy of the document with all sections, see nature.com/documents/nr-reporting-summary-flat.pdf

Life sciences study design

All studies must disclose on these points even when the disclosure is negative.

Sample size	No formal power calculation could be done, due to the lack of knowledge regarding effect size. Sample size was selected based on the number of donors/patients for whom we had gathered data sets.
Data exclusions	No data was excluded
Replication	All results shown were obtained from at least two separate experiments, using cells from different donors. All attempts for replication of the results were successful
Randomization	Randomization was not relevant for this study, since no distinct groups of patients were compared
Blinding	Blinding was not relevant for this study, since no subjective quantification and/or analysis was performed

Reporting for specific materials, systems and methods

We require information from authors about some types of materials, experimental systems and methods used in many studies. Here, indicate whether each material, system or method listed is relevant to your study. If you are not sure if a list item applies to your research, read the appropriate section before selecting a response.

Materials & experimental systems

n/a	Involved in the study	
☐	☒	Antibodies
☒	☐	Eukaryotic cell lines
☒	☐	Palaeontology
☒	☐	Animals and other organisms
☐	☒	Human research participants
☒	☐	Clinical data

Methods

n/a	Involved in the study	
☒	☐	ChIP-seq
☐	☒	Flow cytometry
☒	☐	MRI-based neuroimaging

Antibodies

Antibodies used	Full list of antibodies used for flow cytometry is provided in Supplementary Table 2.<br><br>For microscopy following primary antibodies were used: CD127 (clone eBioRDR5, 14-1278-82, eBioscience, 1:200), HLA-DR (clone L243, 307602, Biolegend, 1:100), CD3e (rabbit, clone EP449E, ab52959, Abcam, 1:50), CD45 (rat, clone YAML501.4, MA5-17687, Invitrogen, 1:100).<br><br>These were detected using following secondary antibodies: goat anti-mouse IgG1 AF488 (catalog # A-21121), goat anti-mouse IgG2a AF647 (catalog # A-21241), goat anti-rat AF680 (catalog # A-21096), goat anti-rabbit AF555 (catalog # A-21428) (all Invitrogen; all 1:200)
Validation	All antibodies used were commercial and validation data can be found on manufacturer's web site. The manufacturer and the product number of each antibody can be found above and in Supplementary Table 2.

Human research participants

Policy information about studies involving human research participants

Population characteristics	Paired peripheral blood and non-affected as well as cancerous gut tissue was obtained from patients undergoing colorectal cancer surgery. Patient information can be found in Supplementary Table 1. Peripheral blood of healthy donors was obtained from the blood bank of Karolinska University Hospital. The age and gender of healthy blood donors is not disclosed and could be m/f/d, 18-60 years old.
Recruitment	Patients were recruited prior to their planned colorectal cancer surgery and written informed consents were obtained from the

Recruitment | patients. In order to be able to obtain cancer tissue biopsy patients recruited had to be diagnosed with Tumor stage > 2, possibly influencing the results of this study. How the selection criteria/bias is likely to impact the experimental results is unclear. Recruitment followed our ethical approval at Karolinska Institutet.

Ethics oversight | Sample collection was approved by the Swedish Ethical Review Authority.

Note that full information on the approval of the study protocol must also be provided in the manuscript.

Flow Cytometry

Plots

Confirm that:

☒ The axis labels state the marker and fluorochrome used (e.g. CD4-FITC).

☒ The axis scales are clearly visible. Include numbers along axes only for bottom left plot of group (a 'group' is an analysis of identical markers).

☒ All plots are contour plots with outliers or pseudocolor plots.

☒ A numerical value for number of cells or percentage (with statistics) is provided.

Methodology

Sample preparation	Peripheral blood mononuclear cells (PBMCs) were isolated from buffy coats, or from whole venous blood of healthy donors. Paired peripheral blood and non-affected as well as cancerous gut tissue was obtained from patients undergoing colorectal cancer surgery. PBMCs were isolated using Ficoll gradient density centrifugation. To isolate intestinal mononuclear cells (MNCs) muscle and adipose tissues were removed and remaining gut tissue was mechanically disrupted, followed by enzymatic digestion with 250 μg/ml DNase and collagenase II at 37°C and magnetic stirring at 450rpm for 45 minutes. Cell suspension was filtered using 70μm cell strainer. Mononuclear cells were isolated using Ficoll gradient density centrifugation.
Instrument	Samples were acquired on BD LSR FortessaTM equipped with 355-, 405-, 488-, 561-, and 639-nm lasers. Cells were sorted using BD FACSAriaTM Fusion Cell Sorter.
Software	Diva software for acquisition. Analysis was performed using FlowJo v. 9.9 (TreeStar).
Cell population abundance	Following FACS sorting of cell populations accounting for under 1 percent of CD45+ cells, cell purity was determined by flow cytometry and was at least 95%
Gating strategy	Gating strategies are shown in Supplementary Figure 1a and Supplementary Figure 7a.

☒ Tick this box to confirm that a figure exemplifying the gating strategy is provided in the Supplementary Information.
